# Observations of membrane fusion in a liposome dispersion: the missing fusion intermediate?

**DOI:** 10.12688/f1000research.6003.2

**Published:** 2015-03-18

**Authors:** Marianna Foldvari

**Affiliations:** 1 School of Pharmacy, Waterloo Institute of Nanotechnology, University of Waterloo, Waterloo, ON N2L 3G1, Canada

**Keywords:** membrane fusion, liposome, fusion mechanism, freeze-fracture, fusion intermediate

## Abstract

Early intermediate structures of liposome-liposome fusion events were captured by freeze-fracture electron microscopic (EM) technique. The images show the morphology of the fusion interface at several different stages of the fusion event. One of the intermediates was captured at a serendipitous stage of two vesicles’ membranes (both leaflets) merging and their contents starting to intermix clearly showing the fusion interface with a previously unseen fusion rim. From the morphological information a hypothetical sequence of the fusion event and corresponding lipid structural arrangements are described.

## Introduction

Phospholipid vesicles (liposomes) are suitable models for studying biological membrane behaviour. Liposomes can be made from a single lipid or lipid mixtures that can provide opportunities to investigate membrane-related events under different conditions and in the presence of additives. The fusion process has been investigated in various artificial membrane systems using electron microscopic, small-angle X-ray scattering (SAXS), nuclear magnetic resonance (NMR) and fluorescence-based kinetic techniques ^1– 5^. Despite the efforts, complete understanding of the molecular structural and kinetic details of the fusion event is still lacking.

The first fusion mechanism from early studies proposed ‘lipidic particles’ (intermediates between lamellar and H _II_ hexagonal phases of the phospholipids or inverted micelles) as the possible intermediate in the fusion process of model lipid vesicles ^6– 8^, since these particles seemed to be present at attachment sites of lipid vesicles and could be visualized by freeze-fracture EM. These intermediates could also be detected in fusion processes of biological membranes (exocytosis, myoblast fusion, protoplast fusion). For example, Satir *et al.* ^9^ observed small particles arranged in rosettes at the site where fusion of the mucocysts in Tetrahymena pyriformis was initiated. Other authors questioned the existence of lipidic particles as dynamic fusion intermediates ^10, 11^ because these particles could not be observed every time at fusion interfaces, and suggested that ‘lipidic particles’ develop subsequent to the fusion process. However, it was suspected that some type of a non-bilayer structure formed in the fusion event. Bearer *et al.* ^10^ specifically suggested the existence of an “’elusive’ intermediate” that has not been visualized in published morphological studies and suggest that the absence of lipidic particles or other intermediate structures may not mean that some dynamic process is taking place and that fusion intermediates could be unstable and convert to different polymorphic forms.

Another mechanism proposed was the ‘stalk mechanism’ ^12– 14^, which involved the formation of a trilaminar structure between the closely apposed bilayers such that the outer monolayers bend to the side to allow joining of the inner monolayers (trans-monolayer contact (TMC) ^15^), which form a stalk at the attachment site of the two membranes ^12, 15– 17^.

The theoretical sequence of events in model lipid membrane fusion can be summarized as follows: 1) close apposition of the two bilayers (<1 nm); 2) local dehydration of phosphorus head groups; 3) destabilization of bilayers; and *either* 4a) formation of inverted micelle intermediates (IMI) at the attachment site; *or* 4b) formation of stalk and TMC); and 5) completion of fusion, i.e. the leakless mixing of contents of two vesicles.

Most of these stages of membrane fusion were described and indirectly measured or modeled, but direct visual evidence is still lacking. Siegel ^18^ and Cullis *et al.* ^19^, and more recently Lentz *et al.* ^20, 21^ speculated that the reason why only some of the actual intermediate structures were detected or visualized is the short lifetime (1 msec or less ^18^) of any given fusion intermediate, making the capture very challenging even with rapid freezing, ^31^P-NMR or SAXS techniques.

In this study, we have observed some fusion intermediate structures in a liposome system in the presence of glycerol by freeze-fracture EM. One of these intermediates, the ‘fusion rim intermediate’ may provide new structural/morphological information on membrane fusion events.

## Methods

Liposomes (small unilamellar vesicles, SUVs) were prepared with soybean lipids (Centrolex P; Central Soya, Fort Wayne, IN). The liposomes were prepared by high shear dispersion using Microfluidizer M110 (Microfluidics Inc. Newton, MA). The liposomes were freeze-fractured without glycerol or after preincubation in 30% v/v glycerol for 30 minutes at room temperature. A drop of the liposome suspension was placed on a gold specimen stub and rapidly frozen in liquid nitrogen cooled Freon 22 (-158°C). All samples were fractured at -105°C in a Balzers 360 freeze-fracture unit. The fracture surfaces were shadowed at 45° angle with a thin layer of platinum-carbon followed by vertical deposition of a carbon layer for replica support. The replicas were floated onto the surface of distilled water and subsequently cleaned with sodium hypochlorite (5% chlorine) and 60% sulfuric acid. After the final washing in distilled water the replicas were picked up on 200 mesh copper grids and examined in a Phillips 200 EM and photographed on Kodak fine grain positive film.

## Results

**Figure 1.**
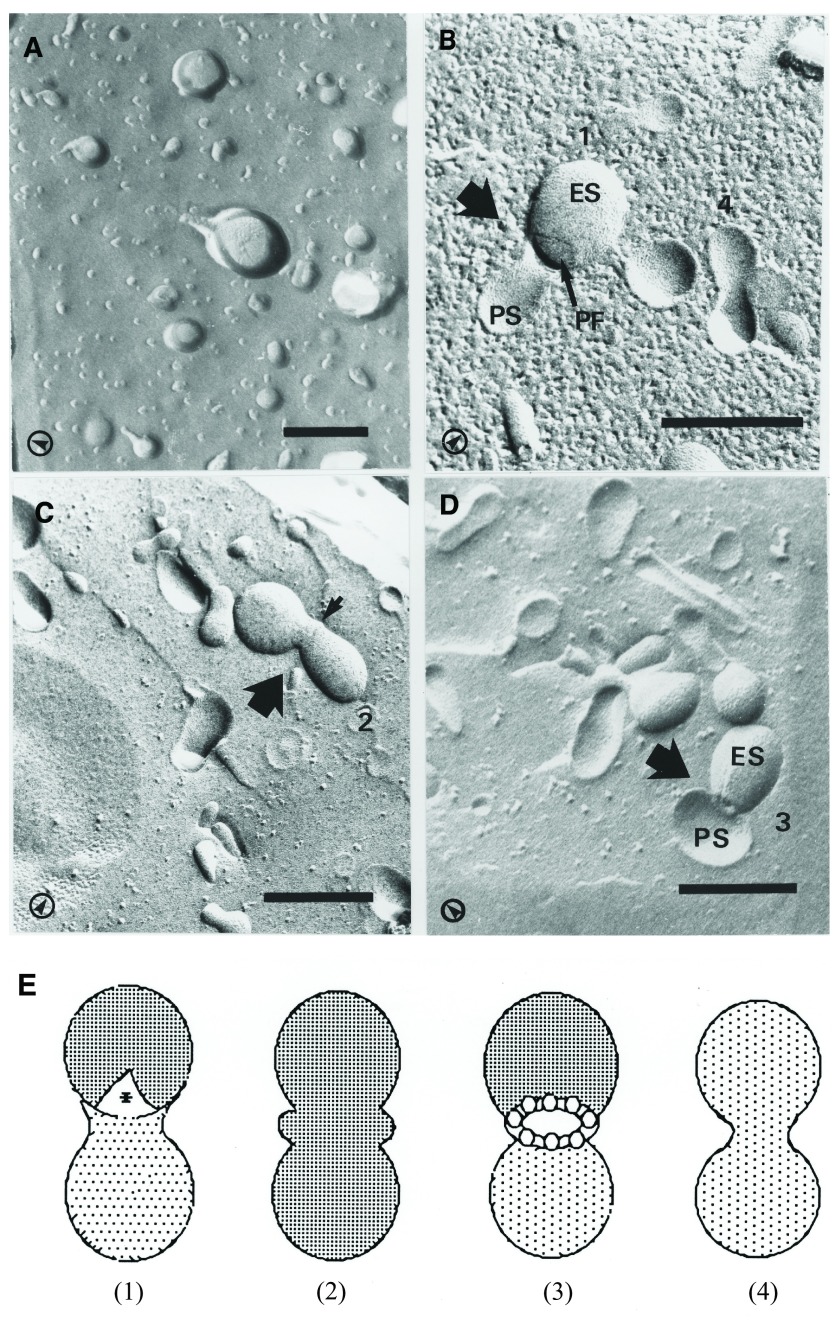
Fusion events in a model liposome dispersion induced by glycerin. Various stages of liposome-liposome fusion event were captured by rapid freezing. **A**) Liposomes were freeze-fractured without the addition of glycerol. **B–D**) Liposomes were preincubated with 30% v/v for 30 minutes before freezing. **B**) The first stage of liposome-liposome fusion: the joining of the outer monolayers of the liposomes (large arrow) is apparent without communication between their internal spaces. **C**) Early liposome-liposome fusion intermediate (large arrow) fractured at the exterior surface of the membrane. The rim between the two vesicles (small arrow) is indicative of non-bilayer intermediate structures. **D**) Late liposome-liposome fusion intermediate (large arrow). One of the liposomes was fractured on the external surface, the other on the internal surface with its internal space is being visible and exposing the fusion interface. The presence of small (10–12 nm) particles can be distinguished at the fusion rim. **E**) Schematic representation of the identified fusion intermediates. Numbers on micrographs correspond to the numbers of the diagrams. Freeze-fracture nomenclature ^27^: ES - external surface; PS - cytoplasmic surface (in this case interior surface of liposome); PF and * - fractured face of the lipid monolayer adjacent to the interior space of the vesicle; arrows in left corners of micrographs indicate the shadowing direction. Bars 250 nm.

**Figure 2.**
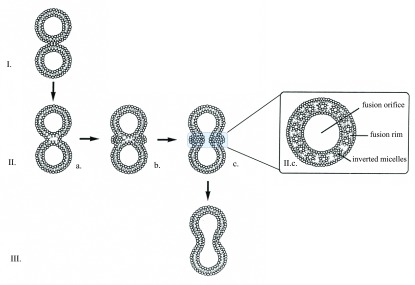
Possible fusion mechanism in a model liposome dispersion induced by glycerin. **I.** Close apposition of two bilayers and formation of an aggregation site when bilayers touch each other. **II.** Merging of two bilayers. **a**) formation of the initial fusion product: the outer leaflets of the bilayers of both vesicles join (fuse), while their inner bilayer leaflets form one common bilayer at the attachment site. **b**) phospholipid molecules from the outer leaflet of the vesicle bilayers, ie. From the attachment site are pushed sideways and form inverted micelles; at the attachment site transient bilayer form composed of the inner bilayer leaflets of the vesicles. **c**) formation of the fusion orifice: the phospholipid molecules from the attachment site are used for the formation of the outer monolayer of the inverted micelles. Inverted micelles are situated all around the fusion orifice. Mixing of the contents of the vesicles has started. Boxed insert shows a horizontal section of the fusion area viewed from above. **III.** Expansion of the bilayer to form a single larger liposome.

The fusion of liposomes was induced by glycerol and several fusion intermediates were captured in the replicas ( Figure 1). Without glycerol, there was no liposome fusion ( Figure 1A). The relatively low concentration of glycerol provided slow dehydration at the phospholipid head group regions which made it possible to observe vesicles still in the fusion process. The micrographs captured vesicles (assumed to be) at various stages of the membrane fusion event ( Figure 1A–C). Figure 1B (large arrow) depicts the initial contact between vesicles – their external monolayers fused but no communication between the two aqueous compartments started. Figure 1D shows a previously unseen moment of liposome-liposome fusion. During the freeze-fracturing procedure one of the liposomes was fractured on the outside surface (it shows the E face), while the other shows the cytoplasmic (P) face (the interior surface of liposome). The leakless intermixing of the aqueous contents of the liposomes had started. At the perimeter of the fusion interface small, 10–12 nm diameter, particles can be distinguished, which probably correspond to the inverted micellar intermediates, so called, lipidic particles. This fusion rim intermediate structure is depicted in the insert of Figure 2. The presence of these lipidic particles could be suspected from another micrograph ( Figure 1C, large arrow), which may represent a preceding intermediate state of fusion.

On the basis of this morphological evidence we constructed a schematic set of drawings to represent a modified model for phospholipid vesicle fusion ( Figure 2). When the bilayers of two separate vesicles are in close apposition ( Figure 2 I), an initial fusion product will form. The outer leaflets of the bilayers of the two vesicles fuse, while their inner leaflets form one common bilayer at the attachment site ( Figure 2 IIa). This stage is followed by the formation of inverted micelles (lipidic particles) around the attachment site ( Figure 2 IIb). The organization of the next fusion intermediate ( Figure 2 IIc and Figure 1D) involves the formation of a fusion orifice. At the perimeter of this orifice, the fusion rim can be seen ( Figure 2, insert), which contains inverted micelles all around. The development of an intermediate like this appears feasible from both energetic and morphological viewpoints, if we take into consideration that the excess phospholipid molecules cleared from the attachment site at this particular stage should be accommodated somewhere, until incorporated into the expanded bilayer of the single larger liposome.

## Discussion and conclusions

Glycerol-induced fusion seen in this study may bear similarities to polyethylene glycol (PEG)-induced fusion ^20, 22^ with dehydration at the liposome attachment site contributing to close contact between the bilayers. It is recognized that fusion is a very dynamic event and certain stages of the fusion are easier to demonstrate than others. Due to the low frequency of vesicle collisions and short lifetime of the actual fusion event and the fact that fusion of vesicles in an aqueous medium is not a synchronous event, visualization of intermediary fusion features on all liposomes in a sample is difficult.

Most of the morphological freeze-fracture studies in the literature show liposomes just before fusion or at the stage already well undergoing fusion. It would be important to clarify events at the stage, where bilayers of the two vesicles are merging and communication between their aqueous spaces begins. The freeze-fracture results in this work supplement those previously reported in the literature and potentially add a new visual image of an intermediate structure to the model of membrane fusion. The initial fusion product ( Figure 2 I) must be very similar to the one proposed by Kozlov and Markin ^12^ on the basis of theoretical considerations. The molecular arrangement of lipids in the ‘fusion rim intermediate’ ( Figure 1D and Figure 2 IIc) could provide an alternative interpretation of the IMI ^8, 23^ or could be considered the next step after the previously described stalk and TMC intermediate ^15, 17^ (this latter may correspond to the image on Figure 1B), and may also be similar to membrane hemifusion events involving proteins ^21, 24– 26^. However, this study has limitation in that the morphological observation and the schematic interpretation are from a single examination and limited to one lipid system and fusogenic agent combination that will require other studies and confirmation in the future.

## References

[1] Marsden HR Tomatsu I Kros A: Model systems for membrane fusion. *Chem Soc Rev.* 2011;40(3): 1572–85. 10.1039/c0cs00115e 21152599

[2] Stark B Pabst G Prassl R: Long-term stability of sterically stabilized liposomes by freezing and freeze-drying: Effects of cryoprotectants on structure. *Eur J Pharm Sci.* 2010;41(3–4):546–55. 10.1016/j.ejps.2010.08.010 20800680

[3] Qian S Wang W Yang L: Structure of transmembrane pore induced by Bax-derived peptide: evidence for lipidic pores. *Proc Natl Acad Sci U S A.* 2008;105(45):17379–83. 10.1073/pnas.0807764105 18987313 PMC2582298

[4] Wilschut J Düzgüneş N Fraley R: Studies on the mechanism of membrane fusion: kinetics of calcium ion induced fusion of phosphatidylserine vesicles followed by a new assay for mixing of aqueous vesicle contents. *Biochemistry.* 1980;19(26):6011–21. 10.1021/bi00567a011 7470445

[5] Lee J Lentz BR: Evolution of lipidic structures during model membrane fusion and the relation of this process to cell membrane fusion. *Biochemistry.* 1997;36(21):6251–9. 10.1021/bi970404c 9174340

[6] Cullis PR Hope MJ Tilcock CP: Lipid polymorphism and the roles of lipids in membranes. *Chem Phys Lipids.* 1986;40(2–4):127–44. 10.1016/0009-3084(86)90067-8 3742670

[7] Verkleij AJ van Echteld CJ Gerritsen WJ: The lipidic particle as an intermediate structure in membrane fusion processes and bilayer to hexagonal H _II_ transitions. *Biochim Biophys Acta.* 1980;600(3):620–4. 10.1016/0005-2736(80)90465-4 7407134

[8] Cullis PR Hope MJ: Effects of fusogenic agent on membrane structure of erythrocyte ghosts and the mechanism of membrane fusion. *Nature.* 1978;271(5646):672–4. 10.1038/271672a0 625336

[9] Satir B Schooley C Satir P: Membrane fusion in a model system. Mucocyst secretion in *Tetrahymena*. *J Cell Biol.* 1973;56(1):153–76. 10.1083/jcb.56.1.153 4629881 PMC2108847

[10] Bearer EL Düzgünes N Friend DS: Fusion of phospholipid vesicles arrested by quick-freezing. The question of lipidic particles as intermediates in membrane fusion. *Biochim Biophys Acta.* 1982;693(1):93–8. 10.1016/0005-2736(82)90474-6 7150597 PMC4646659

[11] Wilschut J Hoekstra D: Membrane fusion: lipid vesicles as a model system. *Chem Phys Lipids.* 1986;40(2–4):145–66. 10.1016/0009-3084(86)90068-X 3742671

[12] Kozlov MM Markin VS: On the theory of membrane fusion. The adhesion-condensation mechanism. *Gen Physiol Biophys.* 1984;3(5):379–402. 6510703

[13] Markin VS Albanesi JP: Membrane fusion: stalk model revisited. *Biophys J.* 2002;82(2):693–712. 10.1016/S0006-3495(02)75432-5 11806912 PMC1301879

[14] Markin VS Kozlov MM Borovjagin VL: On the theory of membrane fusion. The stalk mechanism. *Gen Physiol Biophys.* 1984;3(5):361–77. 6510702

[15] Siegel DP: The modified stalk mechanism of lamellar/inverted phase transitions and its implications for membrane fusion. *Biophys J.* 1999;76(1 Pt 1):291–313. 10.1016/S0006-3495(99)77197-3 9876142 PMC1302519

[16] Chernomordik LV Kozlov MM: Membrane hemifusion: crossing a chasm in two leaps. *Cell.* 2005;123(3):375–82. 10.1016/j.cell.2005.10.015 16269330

[17] Kozlovsky Y Chernomordik LV Kozlov MM: Lipid intermediates in membrane fusion: formation, structure, and decay of hemifusion diaphragm. *Biophys J.* 2002;83(5):2634–51. 10.1016/S0006-3495(02)75274-0 12414697 PMC1302349

[18] Siegel DP: Inverted micellar structures in bilayer membranes. Formation rates and half-lives. *Biophys J.* 1984;45(2):399–420. 10.1016/S0006-3495(84)84164-8 6365189 PMC1434864

[19] Verkleij AJ Leunissen-Bijvelt J de Kruijff B: Non-bilayer structures in membrane fusion. *Ciba Found Symp.* 1984;103:45–59. 10.1002/9780470720844.ch4 6561137

[20] Lentz BR: PEG as a tool to gain insight into membrane fusion. *Eur Biophys J.* 2007;36(4–5):315–26. 10.1007/s00249-006-0097-z 17039359

[21] Lentz BR Siegel DP Malinin V: Filling potholes on the path to fusion pores. *Biophys J.* 2002;82(2):555–7. 10.1016/S0006-3495(02)75420-9 11806900 PMC1301867

[22] Lee J Lentz BR: Outer leaflet-packing defects promote poly(ethylene glycol)-mediated fusion of large unilamellar vesicles. *Biochemistry.* 1997;36(2):421–31. 10.1021/bi9622332 9003195

[23] Hafez IM Cullis PR: Roles of lipid polymorphism in intracellular delivery. *Adv Drug Deliv Rev.* 2001;47(2–3):139–48. 10.1016/S0169-409X(01)00103-X 11311989

[24] Langosch D Crane JM Brosig B: Peptide mimics of SNARE transmembrane segments drive membrane fusion depending on their conformational plasticity. *J Mol Biol.* 2001;311(4):709–21. 10.1006/jmbi.2001.4889 11518525

[25] Hernandez JM Kreutzberger AJ Kiessling V: Variable cooperativity in SNARE-mediated membrane fusion. *Proc Natl Acad Sci U S A.* 2014;111(33):12037–42. 10.1073/pnas.1407435111 25092301 PMC4143004

[26] Tsai HH Chang CM Lee JB: Multi-step formation of a hemifusion diaphragm for vesicle fusion revealed by all-atom molecular dynamics simulations. *Biochim Biophys Acta.* 2014;1838(6):1529–35. 10.1016/j.bbamem.2014.01.018 24468064

[27] Branton D Bullivant B Gilula NB: Freeze-etching nomenclature. *Science.* 1975;190(4209):54–6. 10.1126/science.1166299 1166299

