# Supplementary material for: Observations of membrane fusion in a liposome dispersion: the missing fusion intermediate?
Source: F1000Res. 2015 Mar 18;4:4. Originally published 2015 Jan 8. [Version 2] doi: 10.12688/f1000research.6003.2 (PMC4431385; doi:10.12688/f1000research.6003.2)
Supplement: Peer review history

# Peer review: Referee response for version 2

**DOI:** 10.5256/f1000research.6725.r8030

**Published:** 2015-05-11

**Authors:** James McNew

**Affiliations:** 1 Department of Biosciences, Rice University, Houston, TX, USA

The author correctly acknowledges the limitation of the current approach and has tempered interpretation accordingly.

I have read this submission. I believe that I have an appropriate level of expertise to confirm that it is of an acceptable scientific standard.

# Peer review: Referee response for version 2

**DOI:** 10.5256/f1000research.6725.r8028

**Published:** 2015-04-29

**Authors:** M Joseph Costello

**Affiliations:** 1 Department of Cell Biology and Physiology, University of North Carolina, Chapel Hill, NC, USA

The minimal revision and associated comments did not adequately address the concerns expressed in the review.

I have read this submission. I believe that I have an appropriate level of expertise to state that I do not consider it to be of an acceptable scientific standard, for reasons outlined above.

# Peer review: Referee response for version 2

**DOI:** 10.5256/f1000research.6725.r8029

**Published:** 2015-04-23

**Authors:** Jesse Hay

**Affiliations:** 1 Division of Biological Sciences, The University of Montana, Missoula, MT, USA

This paper reports essentially a single finding with a single system and therefore it is important that it be viewed as motivation for further investigations rather than as strong support for a particular membrane fusion model.  However, I believe the authors have adequately conveyed these limitations in their response to the earlier reviews and in the revised version of the paper.

I have read this submission. I believe that I have an appropriate level of expertise to confirm that it is of an acceptable scientific standard.

# Peer review: Referee response for version 1

**DOI:** 10.5256/f1000research.6424.r7233

**Published:** 2015-01-30

**Authors:** Jesse Hay

**Affiliations:** 1 Division of Biological Sciences, The University of Montana, Missoula, MT, USA

This submission uses freeze-fracture electron microscopy to observe fusion intermediates in glycerol-induced fusion of unilamellar liposomes.  Though fusion in this system is asynchronous, several images are presented that presumably correspond to different sequential intermediates in membrane fusion.  One particularly suggestive image is interpreted to indicate that the hemi-fusion to full-fusion transition proceeds through an intermediate containing a circular array of lipidic particles just beneath the junction between the fused outer leaflets.  The lipid particle model is proposed to potentially reflect the mechanism of membrane fusion induced by biological fusion protein machinery.  The lipid particle model is not a new idea, but has been controversial and lacked direct support.

The paper is interesting and makes a unique contribution to understanding the lipidic intermediates in fusion.  However, it was disappointing that only a single image of the key intermediate is shown and therefore the characteristics of the intermediate cannot be confirmed.  If more examples were analyzed, it should be possible to estimate quantitative parameters such as the approximate number of lipid molecules in the particles, how uniform they are and how often they occur per unit of membrane, etc., that might allow future energetic understanding of the proposed process.  Likewise I found it somewhat disappointing that only glycerol was used to induce fusion.  If the proposed intermediate is generalizable to biological membrane fusion, it should be apparent using another fusogen.

I have read this submission. I believe that I have an appropriate level of expertise to confirm that it is of an acceptable scientific standard, however I have significant reservations, as outlined above.

# Response

**Published:** 2015-03-13

**Authors:** Marianna Foldvari

**Affiliations:** University of Waterloo, Canada

Thank you for the valid comments on the limited number of events in the experiment. In this case we have mostly observed fusion-related events at stages that are not revealing the moment of complete merging of the membranes. Following the field in the past two decades indicate that capturing a fusion event at the most revealing time point is indeed a challenge. Due to the rarity of such image, it is important to present this to the scientific community. As we indicate the serendipity of such finding, it is recognized that this paper can only be a small piece of information that stimulates further studies.

# Peer review: Referee response for version 1

**DOI:** 10.5256/f1000research.6424.r7364

**Published:** 2015-01-22

**Authors:** M Joseph Costello

**Affiliations:** 1 Department of Cell Biology and Physiology, University of North Carolina, Chapel Hill, NC, USA

Conventional freeze fracture experiments were performed on small unilamellar vesicles prepared from soybean lipids using a microfluidizer.  Glycerol was used to induce fusion, which was random producing a few pairs of vesicles in the process of fusion.  The images of intermediate states are interpreted to support the presence of a ring of lipidic particles at the critical point of bilayer-bilayer fusion. This approach is promising and the images are interesting; however, there are major concerns with the controls and with the interpretation of the images.

**Specific comments:**

 

1. Figure 1A.  The image does not display a freeze fracture experiment.  There is no fracture plane through the lipid vesicles.  If such a fracture plane existed in this region, then vesicles with both convex and concave surfaces would be visible (as in subsequent images).  Only convex surfaces are visible.  In addition the convex surface of the larger structures have a revealing pattern of density:  the four larger vesicles have a dark rim and light center.  This is due to the carbon layer that builds up on the curved surface to be thick near the edges but thin enough in the center to be nearly transparent. It appears that a layer of sample, perhaps adjacent to a fractured region, was shadowed to reveal some vesicles but not the expected vesicle size distribution or fracture pattern of a control. 
2. Figure 1B.  The vesicles exposed in this image are consistent with a normal fracture pattern where both convex and concave vesicles are visible.  It should be noted that the fracture step through the monolayer of lipid is present on concave vesicles and, as the authors point out, on the large vesicle where a flap has been fractured away.  The difficulty with this image is that the background ice is not smooth as would be expected for samples prepared in 30% glycerol.  The authors should explain why the background here is different from those in subsequent freeze fracture images.  One possibility is that some etching occurred.
3.  Figure 1C.  The main emphasis in this image is the mound or bump near the fusion interface of the vesicle pair labeled 2.  The interpretation is that the bump represents a fracture through a ring of lipidic particles.  The problem is that a single bump is not consistent with a ring of particles and the hydrophobic plane of fracture around a lipid particle may not appear as a bump or a bulge (as a whole particle might).  Fractures from several different viewpoints and proceeding around the fusion site are needed to properly interpret this pattern in terms of fusion intermediate structures.  A single image showing a slight protrusion is not convincing even if the interpretation turns out to be correct.
4. Figure 1D.  This shows an interesting image of a fusion contact site (labeled 3).  The interpretation of this pattern as a fracture through a ring of lipid particles is difficult to justify based on the irregular size of the particles in the ring.  More such images are needed showing particles of consistent size and distribution.  In addition the image shows some other features that should be explained, such as the large number of oblong (or rod-like) vesicles, up to about five, and the large number of small particles in the background ice (also present in 1C).  It is not clear whether these particles are from contaminant proteins in the solution or from artifacts deposited during the fracturing or shadowing steps. 
5. Figures 1E and 2. The diagrams and molecular interpretations emphasize the role of lipidic particles in the fusion process.  These particles have been implicated in bilayer and membrane fusion for decades without clear resolution about their presence in native fusion events within cells.  The use of glycerol to induce fusion implies that glycerol does not significantly interact with the lipid bilayers or induce lipidic particles.  At least one report suggests that glycerol (unlike polyethylene glycol) can intercalate within the lipid head groups to the extent of inducing interdigitation of lipid chains in the gel phase (BBA 731:97-108, 1983).  Less interaction is seen for fluid phase lipids as used here but glycerol is not a passive inducer of fusion.  Furthermore, Bearer *et al. *(ref. 10) suggest that glycerol induces lipidic particles and that such particles are not typically found in the fusion process in their model system.  The critical point is that the glycerol-induced fusion of soy lipids may not represent natural lipid fusion and thus is not likely to expose the long sought lipid pore intermediate in natural fusion events.

I have read this submission. I believe that I have an appropriate level of expertise to state that I do not consider it to be of an acceptable scientific standard, for reasons outlined above.

# Response

**Published:** 2015-03-13

**Authors:** Marianna Foldvari

**Affiliations:** University of Waterloo, Canada

The author thanks the reviewer for the comments and alternative ideas. The freeze-fracture/etching technique used in this experiment utilized a Balzers 360 instrument which has certain limitations related to manual operation and timing of the sample mounting, temperature adjustment, fracturing and shadowing steps, providing some inconsistencies within sample areas. Nevertheless, the vesicles and fusion events can be observed and some new useful information may be gleaned from this liposome system.

Previous synchrotron x-ray scattering studies examined fusion events in various lipid membrane compositions in aligned multilayer lipid deposit samples [Yang and Huang (2002) Science 297:1877-1879; Yang and Huang (2003) Biophys J. 84:1808-1817; Wang et al. (2006) Biophys. J. 91:736-743 and Qian et al (2008) PNAS 105:17379-83.]  In these studies the electron density distribution was used to map the merging of apposed lipid bilayers and the presence of transmembrane pores and the stalk structure.

However, the scarcity of visual information on lipid vesicle interactions captured at different time points lead us to present these images as possible additional information.

In the paper by Bearer et al. (1982) the authors have examined PE:PS and PC: CL vesicles by quick freezing in the presence of Ca ^2+^ or glycerol and Ca ^2+^. It was stated that no lipidic particles were observed after the addition of Ca ^2+^ and very few vesicles showed lipidic-particle-like structures after preincubation with glycerol followed by Ca ^2+^ addition and this was only after long incubations of 1-2 h, although fusion-related images were frequently observed. We think that the findings in this study do not exclude the possibility of existence of various fusion intermediates just because they were not captured in the samples viewed. The authors actually suggest that the absence of lipidic particles or other intermediate structures may not mean that some dynamic process is taking place and that fusion intermediates could be unstable and convert to different polymorphic forms.

As the authors state: “This conclusion [ie. “lipidic particles (as defined by their morphology in freeze-fracture electron microscopy) are not involved as an intermediate in the stages of fusion”], however, does not exclude the possibility of a transitory intermediate at the site of membrane fusion which involves a lipid conformation distinguishable from the unmodified stable bilayer configuration. This 'elusive' intermediate, which is not visualized at present in any morphological studies, could be an inverted micellar or some other non-bilayer structure or a small domain of a more condensed or crystalline lipid bilayer. For lack of concrete evidence at this point, this 'intermediate' could be characterized simply as a local perturbation of the lipid bilayer structure, which allows mixing of lipid molecules between the two closely apposed membranes.”

While it is agreed that one image is not sufficient to base final conclusions, this note (or ‘case report’) attempts to report an interesting morphological observation that others in the field may find motivating.

We have revised the paper to clarify that the morphological observation and the schematic interpretation are from a single examination and limited to one lipid system and fusogenic agent combination that will require other studies and confirmation in the future.

# Peer review: Referee response for version 1

**DOI:** 10.5256/f1000research.6424.r7365

**Published:** 2015-01-22

**Authors:** James McNew

**Affiliations:** 1 Department of Biosciences, Rice University, Houston, TX, USA

This is an intriguing observation of potential liposomal fusion intermediates.  While the method of inducing protein-free lipid mixing and fusion is unconventional, the structures observed by freeze fracture are unique.  I think it would be important to confirm these intermediates under conditions that have been studied by other means.  The wealth of information on PEG-mediated liposome fusion could be brought to bear if similar conditions were used.  Additionally, it would be beneficial to examine the lipid requirements of such intermediates.  While the lifetime of these structures are likely short making their identification challenging, altered lipid compositions such as at the inclusion of hexagonal phase ii forming lipids like phosphatidylethanolamine may increase the propensity of observing the types of intermediated like the "fusion rim" or "fusion orifice" depicted in this work.  Overall, the proposed intermediate are interesting, but require further experimental evidence to support their role as bona fide fusion intermediates.

I have read this submission. I believe that I have an appropriate level of expertise to confirm that it is of an acceptable scientific standard, however I have significant reservations, as outlined above.

# Response

**Published:** 2015-03-13

**Authors:** Marianna Foldvari

**Affiliations:** University of Waterloo, Canada

The reviewer’s comments are appreciated and will stimulate further work regarding the better understanding of fusion mechanism. It is agreed that the proposed fusion intermediate observed in this liposome system only represents one possible lipid organization at the fusion interface and further studies with the addition of other lipids, proteins or fusogenic agents could provide more clarity of the fusion event.
